# *Mas*-Mediated Antioxidant Effects Restore the Functionality of Angiotensin Converting Enzyme 2-Angiotensin-(1–7)-*Mas* Axis in Diabetic Rat Carotid

**DOI:** 10.1155/2014/640329

**Published:** 2014-04-29

**Authors:** Larissa Pernomian, Mayara Santos Gomes, Carolina Baraldi Araujo Restini, Ana Maria de Oliveira

**Affiliations:** ^1^Faculdade de Ciencias Farmacêuticas de Ribeirão Preto, Universidade de São Paulo, Avenida do Café s/n, 14040-903 Ribeirão Preto, SP, Brazil; ^2^Faculdade de Medicina, Universidade de Ribeirão Preto, Avenida Costábile Romano 2201, 14096-900 Ribeirão Preto, SP, Brazil

## Abstract

We hypothesized that endothelial AT_1_-activated NAD(P)H oxidase-driven generation of reactive oxygen species during type I-diabetes impairs carotid ACE2-angiotensin-(1–7)-*Mas* axis functionality, which accounts for the impaired carotid flow in diabetic rats. We also hypothesized that angiotensin-(1–7) chronic treatment of diabetic rats restores carotid ACE2-angiotensin-(1–7)-*Mas* axis functionality and carotid flow. Relaxant curves for angiotensin II or angiotensin-(1–7) were obtained in carotid from streptozotocin-induced diabetic rats. Superoxide or hydrogen peroxide levels were measured by flow cytometry in carotid endothelial cells. Carotid flow was also determined. We found that endothelial AT_1_-activated NAD(P)H oxidase-driven generation of superoxide and hydrogen peroxide in diabetic rat carotid impairs ACE2-angiotensin-(1–7)-*Mas* axis functionality, which reduces carotid flow. In this mechanism, hydrogen peroxide derived from superoxide dismutation inhibits ACE2 activity in generating angiotensin-(1–7) seemingly by activating *I*_Cl,SWELL_, while superoxide inhibits the nitrergic *Mas*-mediated vasorelaxation evoked by angiotensin-(1–7). Angiotensin-(1–7) treatment of diabetic rats restored carotid ACE2-angiotensin-(1–7)-*Mas* axis functionality by triggering a positive feedback played by endothelial *Mas* receptors, that blunts endothelial AT_1_-activated NAD(P)H oxidase-driven generation of reactive oxygen species. *Mas*-mediated antioxidant effects also restored diabetic rat carotid flow, pointing to the contribution of ACE2-angiotensin-(1–7)-*Mas* axis in maintaining carotid flow.

## 1. Introduction

Vascular dysfunction triggered by type I-diabetes has been extensively described as an important risk factor for the development of carotid atherosclerosis in the genesis of cerebrovascular diseases, such as stroke [1–4]. The major mechanisms underlying diabetic vascular dysfunction result from changes in the functionality of the main systems involved in the control of arterial tonus [5–9], such as renin-angiotensin system (RAS). Indeed, the upregulation of angiotensin converting enzyme- (ACE-) angiotensin II-AT_1_ axis from vascular RAS seems to play a crucial role in the pathogenesis of diabetic vascular dysfunction and complications. Yousif et al. [5, 6] and Pernomian et al. [8, 9] showed that type I-diabetes enhances the vasocontractile response evoked by angiotensin II [5, 6, 8, 9], which damages vascular function and contributes with atherogenesis by affecting both the vascular tone and the progression of vascular inflammation [10]. Moreover, angiotensin converting enzyme (ACE) inhibitors or AT_1_ antagonists attenuate carotid atherosclerosis during diabetes by improving vascular function [11–13].

Despite the aggressive effects assigned to ACE-angiotensin II-AT_1_ axis on vascular function during diabetic conditions, there is another important axis from RAS, namely, ACE2-angiotensin-(1–7)-*Mas* axis, that triggers opposite effects to those produced by the former [14, 15]. In this alternative axis, ACE homologue (ACE2) hydrolyzes angiotensin II into angiotensin-(1–7) [16, 17], which is the endogenous ligand of* Mas* receptors [18]. In vessels, such as rat carotid, the activation of* Mas* receptors evokes a nitrergic relaxation [19] that has been correlated with vasoprotective effects in diabetic conditions [20]. This perspective has pointed the vascular ACE2-angiotensin-(1–7)-*Mas* axis as a potential therapeutic target to attenuate diabetic endothelial dysfunction and the subsequent vascular complications. Nevertheless, the vasoprotective therapeutic efficacy of drugs aimed at the activation of ACE2-Angiotensin-(1–7)-*Mas* axis depends on the integrity of the functionality of this axis during the disease. To the best of our knowledge, there are no evidences concerning the consequences of type I-diabetes on the functionality of vascular ACE2-angiotensin-(1–7)-*Mas* axis. However, our previous findings suggest an indirect evidence concerning these consequences: in rat carotid, type I-diabetes shifts the Gaussian-like shape of angiotensin II-evoked contraction curve into a sigmoidal shape, due to endothelial AT_1_-activated NAD(P)H oxidase-driven generation of superoxide (O_2_^−^), hydrogen peroxide (H_2_O_2_), and hydroxyl radical (∙OH) [8]. In turn, the Gaussian-like shape of angiotensin II-induced contraction curve in rat carotid results from the relaxation triggered by micromolar concentrations of angiotensin II, which is mediated by* Mas *receptors [19] probably upon the hydrolysis of angiotensin II into angiotensin-(1–7). Thus, our previous findings [8] allow us to hypothesize that endothelial AT_1_-activated NAD(P)H oxidase-driven generation of reactive oxygen species during type I-diabetes impairs the functionality of ACE2-angiotensin-(1–7)-*Mas* axis in rat carotid by inhibiting both the hydrolysis of angiotensin II into angiotensin-(1–7) and the nitrergic signaling pathway underlying* Mas* receptors activation. Considering the vasoprotective effects assigned to vascular ACE2-angiotensin-(1–7)-*Mas* axis [20], we also hypothesize that the impairment of the functionality of this axis would contribute to damage carotid blood flow and resistance in type I-diabetic rats. Furthermore, since the activation of* Mas *receptors inhibits NAD(P)H oxidase-driven generation of reactive oxygen species in endothelial cells [21], we expect that the chronic treatment of type I-diabetic rats with angiotensin-(1–7) would restore the functionality of carotid ACE2-angiotensin-(1–7)-*Mas* axis and carotid blood flow and resistance by restoring both the local hydrolysis of angiotensin II into angiotensin-(1–7) and the nitrergic signaling pathway underlying* Mas* receptors activation.

## 2. Material and Methods

The experiments were carried out in accordance with the Guide for the Care and Use of Laboratory Animals. A prior approval was granted by the Animal Ethics Committee of the Faculty of Medicine from Ribeirão Preto (FMRP) from the University of São Paulo (USP) in Brazil (approval reference number: 007/2009). Male Wistar rats (*Rattus norvegicus*) were used in this study. These animals were kept under a 12 light/12 dark cycle (light from 06:00 to 18:00 h) and fed with regular chow and water* ad libitum*.

### 2.1. Experimental Design and Animal Groups

Type I-diabetes was induced by a single intraperitoneal injection of streptozotocin (STZ, 55 mg/kg) dissolved in citrate buffer (0.09 mol/L, pH 4.5) (day 0) in eight-week-old male Wistar rats (350–400 g). The control group was composed by age-matched normoglycaemic rats that underwent to citrate buffer injection. Fasting glucose levels were determined from rat tail blood samples prior to and 48 h (day 2) after STZ or vehicle injection, by using a one-touch glucometer (LifeScan Inc., Milpitas, CA, USA). Diabetic rats presented glycaemia higher than 300 mg/dL (Table 1). Six weeks after STZ or vehicle injection (day 42), the animals underwent to body weight measurement (Table 2), then they were sacrificed, and the experiments were performed [8, 9]. In some protocols, STZ- or vehicle-treated rats (eight-weeks old) were chronically treated with intraperitoneal daily injections of the selective ACE2 inhibitor DX600 (5 *μ*g/kg/day) or with daily intraperitoneal injections of the selective* Mas* receptors agonist angiotensin-(1–7) (576 *μ*g/kg/day), combined or not with the selective* Mas* receptors antagonist A779 (1 mg/kg/day), for six weeks [22]. All these treatments started at the time of STZ or vehicle injection.

### 2.2. *Ex Vivo* Arterial Reactivity Studies

The functionality of vascular ACE2-angiotensin-(1–7)-*Mas* axis was studied by functional assays of angiotensin II or angiotensin-(1–7) cumulative concentration-response relaxant curves, obtained in* ex vivo* arterial reactivity studies in carotid rings from control or diabetic rats.

#### 2.2.1. Carotid Rings Preparation

Rats were sacrificed by abdominal aortic exsanguination and common carotid arteries were isolated. Carotid rings (4 mm) were placed in 5.0 mL of Krebs-Henseleit bicarbonate buffer (composition in mmol/L: NaCl 118.4; KCl 4.7; CaCl_2_ 1.9; KH_2_PO_4_ 1.2; MgSO_4_·7H_2_O 1.2; NaHCO_3_ 25; C_6_H_12_O_6_ 11.6) in organ bath chambers, gassed with 95% O_2_ and 5% CO_2_ and maintained at 37°C and pH 7.4. The rings were connected to an isometric force transducer (Letica Scientific Instruments, Barcelona, Spain) to measure changes in the isometric tension [8, 9, 23, 24]. After 60 min of stabilization at a resting tension of 1 g, carotid rings viability was tested with the appropriate molar concentration of phenylephrine that produces 50% of the maximum contraction response (EC_50_) in each experimental group (phenylephrine EC_50_ = 0.1 *μ*mol/L for control rat carotid or phenylephrine EC_50_ = 10 nmol/L for diabetic rat carotid), as previously determined by cumulative concentration-response curves for phenylephrine. The endothelial integrity was verified with the appropriate molar concentration of acetylcholine that produces 100% of the maximum relaxation response (EC_100_) in each experimental group (acetylcholine EC_100_ = 1.0 *μ*mol/L for control rat carotid or acetylcholine EC_100_ = 100 *μ*mol/L for diabetic rat carotid), over phenylephrine-induced precontraction [8, 9]. For studies with endothelium-intact vessels, the ring was discarded if the maximum relaxation induced by acetylcholine did not reach 80–100%. When necessary, the endothelium was mechanically removed by gentle rubbing the vessel with a thin wire. Endothelium was deemed absent when the relaxation response to acetylcholine did not occur [8, 9, 23, 24].

#### 2.2.2. Experimental Protocols

*(1) Cumulative Concentration Response Relaxant Curves for Angiotensin II.* The functionality of vascular ACE2 (i.e., the activity of vascular ACE2 in generating angiotensin-(1–7)) was studied by relaxant curves induced by angiotensin II in rat carotid. This approach was supported by two evidences: (1) the angiotensin 1–7* Mas* receptor antagonist, A779, partially inhibits the relaxation evoked by angiotensin II in rat carotid [19], suggesting that angiotensin II is converted into angiotensin-(1–7) in this bed; and (2) the unique enzyme that is able to convert angiotensin II into angiotensin-(1–7) in vascular tissues is ACE2 [17, 25].

Relaxation cumulative concentration-response curves for angiotensin II (10^−9^–10^−4^ mol/L) were obtained in endothelium-intact or endothelium-denuded carotid rings from control or diabetic rats, over the precontraction induced by the appropriate molar concentration of phenylephrine that produces 80% of the maximum contraction response (EC_80_) in each experimental group (phenylephrine EC_80_ = 1.0 *μ*mol/L for control rat carotid or phenylephrine EC_80_ = 0.1 *μ*mol/L for diabetic rat carotid). In this study, we have chosen phenylephrine EC_80_ instead of EC_50_ due to the higher magnitude of the precontraction evoked by the former concentration (too much closer to the maximum contraction). This avoids significant changes on the precontraction value upon pharmacological intervention. In functional assays, this is an important approach, since it avoids significant interferences from the precontraction magnitude variation on the relaxant response magnitude. Finally, EC_80_ can be safely used to induce a precontraction because it is as accurate for the homogeneous distribution of date as the EC_50_, since both of them can be found at the central linear region of sigmoidal curves. The relaxant curves for angiotensin II were obtained in carotid rings from nontreated control or diabetic rats, in the absence or presence of DX600 (10 *μ*mol/L, 30 min) [26], A779 (5.0 *μ*mol/L, 30 min) [19] or the selective AT_2_ antagonist, PD123,319 (0.5 *μ*mol/L, 30 min) [19]. As posteriorly described in the Results section, this protocol confirms that ACE2 converts angiotensin II into angiotensin-(1–7), which in turn partially mediates the relaxation evoked by angiotensin II by activating* Mas* receptors. Thus, this protocol validates the functional studies of angiotensin II-evoked relaxant curves in rat carotid as a method to study vascular ACE2 functionality.

To verify if the reactive oxygen species derived from AT_1_-activated NAD(P)H oxidase impair the functionality of ACE2 in diabetic rat carotid, angiotensin II-induced relaxant curves were obtained in the absence or presence of the selective AT_1_ antagonist, losartan (1.0 *μ*mol/L, 30 min) [27], the selective NAD(P)H oxidase inhibitor, apocynin (0.1 mmol/L, 30 min) [8, 23], the selective O_2_^−^ scavenger, tiron (0.1 mmol/L, 30 min) [8, 23], or the selective H_2_O_2_ scavenger, PEG-catalase (250 U/mL, 30 min) [8, 23]. Also, we investigated the hypothesis that the reactive oxygen species-mediated activation of volume-sensitive Cl^−^ current (*I*_Cl,SWELL_) impairs ACE2 functionality in diabetic rat carotid, by obtaining angiotensin II-evoked relaxant curves in the presence of the selective inhibitor of *I*_Cl,SWELL_, DCPIB (10 *μ*mol/L, 10 min) [28, 29]. This hypothesis was suggested based on the following evidences: (1) the cleavage of angiotensin II by ACE2 is reduced by increasing the physiological extracellular levels of Cl^−^ [30]; and (2) H_2_O_2_ generated from NAD(P)H oxidase-derived O_2_^−^ can activate *I*_Cl,SWELL_ in vascular smooth muscle cells, leading to the efflux of Cl^−^ and thus increasing Cl^−^ extracellular levels [31, 32].

In order to verify if the* Mas*-mediated effects against AT_1_-activated NAD(P)H oxidase-driven generation of reactive oxygen species restore the functionality of ACE2 in diabetic rat carotid, the relaxation curves for angiotensin II were obtained in carotid rings from control or diabetic rats that were chronically treated with angiotensin-(1–7). This protocol was not repeated in carotid rings from rats treated with angiotensin-(1–7) combined with A779 because angiotensin II-evoked relaxation is partially mediated by* Mas* receptors [19].

*(2) Cumulative Concentration-Response Relaxant Curves for Angiotensin-(1–7).* The functionality of vascular* Mas* receptors (i.e., nitrergic vasorelaxant response evoked by angiotensin-(1–7) upon* Mas *receptors activation) was studied by relaxation cumulative concentration-response curves for angiotensin-(1–7) (10^−11^–10^−5^ mol/L), that were obtained in endothelium-intact or endothelium-denuded carotid rings from control or diabetic rats. In these protocols, carotid rings were also precontracted with phenylephrine EC_80_ (1.0 *μ*mol/L for control rat carotid or 0.1 *μ*mol/L for diabetic rat carotid), due to the reasons previously described.

The mediators of angiotensin-(1–7)-induced relaxation were characterized by obtaining these curves in carotid rings from nontreated control or diabetic rats, in the absence or presence of A779 (5.0 *μ*mol/L, 30 min), PD123,319 (0.5 *μ*mol/L, 30 min), the selective nitric oxide (NO) scavenger hydroxocobalamin (0.1 mmol/L, 30 min) [33], the nonselective NOS inhibitor L-NNA (0.1 mmol/L, 30 min), the selective neuronal NOS (*n*NOS) inhibitor L-NPA (50 nmol/L, 30 min), or the selective inducible NOS (*i*NOS) inhibitor 1400 W (10 nmol/L, 30 min) [9].

To verify if the reactive oxygen species derived from AT_1_-activated NAD(P)H oxidase impair the functionality of angiotensin-(1–7)-*Mas* receptors in diabetic rat carotid, angiotensin-(1–7)-induced relaxant curves were obtained in the absence or presence of losartan (1.0 *μ*mol/L, 30 min), apocynin (0.1 mmol/L), tiron (0.1 mmol/L), or PEG-catalase (250 U/mL). We also investigated if the reactive oxygen species-activated *I*_Cl,SWELL_ impairs the functionality of angiotensin-(1–7)-*Mas* receptors in diabetic rat carotid, by obtaining angiotensin II-evoked relaxant curves in the presence of DCPIB (10 *μ*mol/L, 10 min).

In order to verify if the* Mas*-mediated effects against AT_1_-activated NAD(P)H oxidase-driven generation of reactive oxygen species restore the functionality of angiotensin-(1–7)-*Mas* receptors in diabetic rat carotid, the relaxation curves for angiotensin II were obtained in carotid rings from control or diabetic rats that were chronically treated with angiotensin-(1–7). This protocol was not repeated in carotid rings from rats treated with angiotensin-(1–7) combined with A779 because angiotensin-(1–7)-evoked relaxation is mediated by* Mas* receptors [19].

### 2.3. Flow Cytometry in Endothelial Cells

To confirm the functional evidences that the* Mas*-mediated effects induced by the chronic treatment with angiotensin-(1–7) on ACE2-angiotensin-(1–7)-*Mas* axis functionality in diabetic rat carotid involve antioxidant actions against endothelial AT_1_-activated NAD(P)H oxidase-driven generation of reactive oxygen species (O_2_^−^ and H_2_O_2_), flow cytometry assays were performed in carotid-derived endothelial cells, loaded with selective probes for reactive oxygen species.

Carotid arteries were isolated after abdominal aortic exsanguination. Thus, the arteries were longitudinally sectioned and endothelial cells were mechanically isolated by gentle friction with a plastic stem in plates containing Hanks' solution (composition in mmol/L: CaCl_2_ 1.6; MgSO_4_ 1.0; NaCl 145.0; KCl 5.0; NaH_2_PO_4_ 0.5; dextrose 10.0; HEPES 10.0) at pH 7.4. The cell suspensions were centrifuged at 1.375 hg for 5 min, and the pellets were resuspended in 0.5 mL of Hanks's solution in a humidified incubator at 37°C until use [8, 9, 23, 24, 34]. Each *n* comprised a pool of six carotid arteries. The cell viability was previously determined by trypan blue staining (2%) and counting in a Neubauer chamber (Weber Scientific International, Germany).

The endothelial cells were loaded with the nonselective fluorescent dye for reactive oxygen species, dihydroethidium (DHE, 2.5 *μ*mol/L, 20 min, 37°C) [8, 23] or with the selective fluorescent dye for H_2_O_2_, 5(6)-carboxy-2′,7′-dichlorofluorescein diacetate (CDCF-DA, 1 *μ*mol/L, 20 min, 25°C) [9, 35].

The basal levels of O_2_^−^ from endothelial cells isolated from carotid arteries of nontreated control or diabetic rats were measured in the absence or presence of tiron (0.1 mmol/L, 30 min), apocynin (0.1 mmol/L, 30 min), losartan (1.0 *μ*mol/L, 30 min), or DCPIB (10 *μ*mol/L, 10 min), added to the cellular samples during DHE loading. The basal levels of O_2_^−^ were also measured in endothelial cells from carotid arteries of control or diabetic rats chronically treated with angiotensin-(1–7), combined or not with A779.

The basal levels of H_2_O_2_ from endothelial cells isolated from carotid arteries of nontreated control or diabetic rats were measured in the absence or presence of PEG-catalase (3,000 U/mL, 30 min) [36], tiron (0.1 mmol/L, 30 min), apocynin (0.1 mmol/L, 30 min), losartan (1.0 *μ*mol/L, 30 min), or DCPIB (10 *μ*mol/L, 10 min), added to the cellular samples during CDCF-DA loading. The basal levels of H_2_O_2_ were also measured in endothelial cells from carotid arteries of control or diabetic rats chronically treated with angiotensin-(1–7), combined or not with A779.

### 2.4. Immunohistochemical Assays

The expression of ACE2 and* Mas* receptors, as well as the levels of angiotensin-(1–7), was assessed by immunohistochemical assays. These data were correlated with the functionality of ACE2-angiotensin-(1–7)-*Mas* axis in nontreated control or diabetic rat carotid arteries. The effects of the chronic treatment with angiotensin-(1–7) on ACE2 and* Mas* receptors expression in control or diabetic rat carotid were also studied. In turn, the expression of angiotensin-(1–7) was not assessed in carotid arteries from control or diabetic rats that were chronically treated with angiotensin-(1–7), since this treatment tends to increase the vascular levels of the heptapeptide. Furthermore, to confirm the functional evidences concerning the generation of angiotensin-(1–7) by ACE2 in rat carotid and the impairment of ACE2 activity in generating angiotensin-(1–7) in diabetic rat carotid, angiotensin-(1–7) levels were also assessed in carotid arteries from nontreated or DX600-treated control or diabetic rats.

As we aimed to evaluate the expression of ACE2, angiotensin-(1–7) and* Mas* receptors in each vascular layer, mainly in the endothelium, we have chosen immunohistochemistry instead of western blotting (WB) assays, since it is not viable to isolate endothelial cells from rat carotid to perform WB (each *n* of endothelial cells sample would comprise more than 20 carotid arteries to yield a sufficient protein level). In addition, angiotensin-(1–7) levels could not be evaluated by western blotting assays since the molecular weight of angiotensin-(1–7) is lesser than 2.0 kDa, what precludes the use of the available polyacrylamide or tricine gels and molecular weight markers.

After abdominal aortic exsanguination, carotid arteries were isolated and included in paraffin. Ion paraffin-included carotid rings were cut into 3 *μ*m sections and mounted on poly-L-lysine-coated slides, which were rinsed with phosphate-buffered saline (PBS) and immersed in 3% H_2_O_2_ for 20 min to block endogenous peroxidase. Nonspecific protein binding was blocked with normal serum for 30 min [8, 9]. The sections were incubated with the primary antibody against ACE2 (1 : 100) [37],* Mas* receptors (1 : 250) [38], or angiotensin-(1–7) (1 : 100) [39] for 2 h at 25°C. Following washes in PBS, biotinylated pan-specific universal secondary antibody (1 : 300) was applied for 30 min. The slides were incubated with avidin-biotin peroxidase complex for 30 min. They were then counterstained by haematoxylin, dehydrated, and mounted with Permount. As negative controls, all specimens were incubated with an isotope-matched control antibody under identical conditions. The immunolabeling was considered positive when distinct red nuclear or cytoplasmic staining was homogenously present [8, 9].

### 2.5. Carotid Blood Flow Measurement

In order to verify if the functionality of local ACE2-angiotensin-(1–7)-*Mas* axis contributes to the control of carotid blood flow and resistance and if the* Mas*-mediated effects restore carotid blood flow and resistance in diabetic rats, these experiments were performed in control or diabetic rats that were chronically treated or not with DX600, A779 or with angiotensin-(1–7), combined or not with A779.

Rats were anaesthetized with ketamine (50 mg/kg) and xylazine (10 mg/kg), by intraperitoneal injection. The left and right common carotid arteries were exposed. A noninvasive transit-time flow probe (model 1.5RB; Transonic Systems, Inc., Ithaca, NY, USA) was placed around each carotid artery and connected to a flow meter (model T-206; Transonic system, USA). Basal mean blood pressure was determined in anaesthetized rats after femoral artery cannulation [9, 40]. Blood pressure and carotid flow were simultaneously measured after the slow infusion of normal saline (0.9%, 1 mL), that was used as volume expander, in order to prevent the eventual interferences related to the reduced tissue perfusion volume in diabetic rats, whose body weight is reduced (Table 2).

### 2.6. Data Analysis

Data were expressed as the mean ± S.E.M., and the differences between the mean values were assessed by one-way ANOVA and Bonferroni* post-hoc*. The significance level considered was 0.05. We chose one-way ANOVA as the statistical test since there was only one independent variable in the experimental design (i.e., the* treatment* factor), which was divided into two categorical (nominal) variables: (1) the* in vivo* treatment of rats with STZ, citrate buffer, DX600, angiotensin-(1–7), and/or A779; and (2) the* in vitro* pretreatment of carotid rings or carotid endothelial cells with DX600, A779, PD123,319, losartan, hydroxocobalamin, apocynin, tiron, PEG-catalase, or DCPIB. Endothelial removal is also considered as an* in vitro* pretreatment variable, since the removal of endothelial modulation on vascular responses can be equally reached by mechanical or chemical induction.

In the functional studies, relaxant responses were recorded as reductions in the muscular tone evoked by the preconstrictor agent and expressed as grams of tension (absolute relaxation values) on negative scale. The concentration-response curves were fitted using a nonlinear interactive fitting program (GraphPad Prism 5.00; GraphPad Software Inc., San Diego, CA). The maximum effect (*E*_max⁡_⁡) of AngII or Ang-(1–7) was obtained from the nonlinear regression of the agonist curve.

In the flow cytometry analysis, the median values of the fluorescence intensity (FI) emitted by endothelial cells were determined using DIVA software and expressed in fluorescence units (U).

The percentage of the stained area was determined by ImageJ Program (1.46r, Wayne Rasband, NIH, USA) in immunohistochemical assays, in which it was delimited an area of 900 *μ*m^2^ for the media or an area of 45 *μ*m^2^ for the endothelium from carotid sections.

In the* in vivo *experiments, the mean carotid blood flow (*F*) was recorded by 10 min in a computational acquisition system (Dataq, USA), which provided an actual volume flow measurement in a resolution of 0.05 mL/min. Mean blood pressure (BP) baseline values were calculated as the average of the 10 min recording, by using the acquisition system (Dataq, USA).* F* and BP were used to calculate the carotid resistance (*R*) by applying the formulae* R* = BP/*F*.

### 2.7. Drugs, Chemical Reagents, and Other Materials

They are STZ, angiotensin II, angiotensin-(1–7), losartan, PD123,319, hydroxocobalamin, L-NNA, 1400 W, apocynin, tiron, PEG-catalase, CDCF-DA, and DCPIB (Sigma, St. Louis, Mo., USA); DX600 (Anaspec Inc., Fremont, CA, USA); A779 (Bachem California Inc., Torrance, CA, USA); L-NPA (Tocris, Avonmouth, UK); DHE (Invitrogen, Carlsbad, CA, USA); ketamine (União Química, Jabaquara, SP, Brazil); xylazine (Calier Laboratory, Jubatuba, MG, Brazil); rabbit polyclonal anti-ACE2 antibody (Abcam, Cambridge, MA, USA); rabbit anti-angiotensin-(1–7) antibody (Phoenix Pharmaceuticals Inc., Burlingame, CA, USA); rabbit polyclonal anti-angiotensin-(1–7)-*Mas* receptor antibody (Alomone Labs, Jerusalem, Israel); biotinylated universal secondary antibody, avidin-biotin peroxidase complex (Vectastain Elite ABC kit, Universal, Vector Laboratories Inc. U.S. Headquarters, Burlingame, CA, USA).

## 3. Results

### 3.1. Angiotensin II-Induced Relaxation

**Figure 1:**
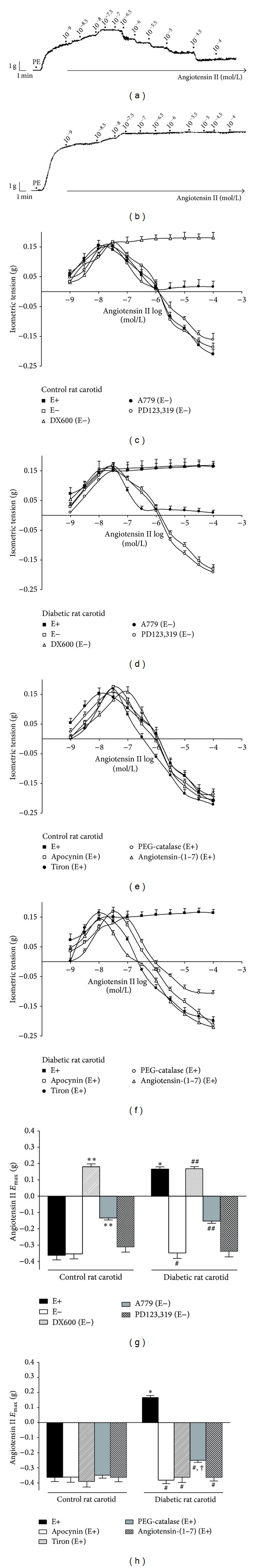
Concentration-response curves for angiotensin II in endothelium-intact (E+) or -denuded (E−) carotid rings from control or diabetic rats, over the precontraction induced by phenylephrine (PE). Representative traces from angiotensin II-evoked relaxation in E+ control (a) or diabetic (b) rat carotid. Effect of the* in vitro* pretreatment with DX600, A779, or PD123,319 in carotid rings from control (c) or diabetic (d) rats. Effect of the* in vitro *pretreatment with apocynin, tiron, PEG-catalase, or the chronic (*in vivo*) treatment with angiotensin-(1–7) in carotid rings from control (e) or diabetic (f) rats. Angiotensin II *E*_max⁡_ in carotid arteries from control or diabetic rats before or after the* in vitro *pretreatment with DX600, A779, PD123,319 (g), apocynin, tiron, or PEG-catalase or the chronic treatment (*in vivo*) with angiotensin-(1–7) (h). The values are significantly different (*P* < 0.01; *n* = 9) from nonpretreated E+ (∗) or E− (∗∗) carotid rings from nontreated control rats, from nonpretreated E+ (#) or E− (##) carotid rings from nontreated diabetic rats, or from PEG-catalase pretreated E+ (†) carotid rings from nontreated control rats.

Phenylephrine EC_80_ evoked a precontraction of 0.29 ± 0.021 g (*n* = 9) in endothelium-intact carotid rings from nontreated control rats. In these vessels, angiotensin II produced a biphasic response over phenylephrine-induced precontraction, which was characterized by a residual contraction (0.15 ± 0.019 g, *n* = 9) evoked by nanomolar concentrations of angiotensin II, followed by a relaxant response (*E*_max⁡_ = −0.36 ± 0.028 g, *n* = 9) induced by micromolar concentrations of angiotensin II (Figures 1(a) and 1(c)). Endothelium removal did not alter the magnitude of phenylephrine-induced precontraction (0.34 ± 0.032 g, *n* = 9) or the relaxant response evoked by angiotensin II (*P* > 0.05*, one-way ANOVA, Bonferroni post-hoc*). In endothelium-denuded carotid rings from nontreated control rat, this relaxant response was completely inhibited by DX600 but partially inhibited by A779 (Figures 1(c) and 1(g)). Losartan inhibited the residual contraction evoked by angiotensin II and thus reduced the maximum relaxation triggered by angiotensin II in endothelium-intact carotid rings from nontreated control rat (Figures 3(a) and 3(c)). The magnitude of phenylephrine-induced precontraction in control rat carotid rings was not altered by DX600 (0.35 ± 0.031 g, *n* = 9), A779 (0.32 ± 0.025 g, *n* = 9), or losartan (0.29 ± 0.023 g, *n* = 9) (*P* > 0.05*, one-way ANOVA, Bonferroni post-hoc*).

Phenylephrine EC_80_ evoked a precontraction of 0.51 ± 0.033 g (*n* = 9) in endothelium-intact carotid rings from nontreated diabetic rats. This value was significantly higher than that one evoked in endothelium-intact carotid rings from nontreated control rats (*P* < 0.001*, one-way ANOVA, Bonferroni post-hoc*). In carotid artery from nontreated diabetic rat, nanomolar concentrations of angiotensin II evoked a persistent residual contraction (0.17 ± 0.014 g, *n* = 9) over phenylephrine-induced precontraction (Figures 1(b) and 1(d)). Diabetes abrogated the relaxant response induced by micromolar concentrations of angiotensin II in rat carotid, which was completely restored by endothelium removal (this result required that the characterization of the mediators of angiotensin II-induced relaxation was performed in endothelium-denuded carotid rings from diabetic rats, as well as from control rats, to compare the responses). In endothelium-denuded carotid rings from nontreated diabetic rats, the relaxant response evoked by angiotensin II was completely inhibited by DX600 but partially inhibited by A779 (Figures 1(d) and 1(g)). Losartan inhibited the residual contraction induced by angiotensin II but allowed micromolar concentrations of angiotensin II to elicit a relaxant response in endothelium-intact carotid rings from nontreated diabetic rat (Figures 3(a) and 3(c)). The magnitude of phenylephrine-induced precontraction in carotid rings from nontreated diabetic rats was not altered by endothelium removal (0.46 ± 0.041 g, *n* = 9) or the pretreatment with DX600 (0.49 ± 0.037 g, *n* = 9), A779 (0.47 ± 0.028 g, *n* = 9), or losartan (0.52 ± 0.045 g, *n* = 9), when compared to nonpretreated endothelium-intact diabetic rat carotid (*P* > 0.05*, one-way ANOVA, Bonferroni post-hoc*).

In endothelium-denuded carotid rings from nontreated control or diabetic rats, the maximum relaxation induced by angiotensin II in the presence of A779 had the same extent (in absolute values of g of tension) as the maximum contraction evoked by angiotensin II in these arteries (Figures 1(c), 1(d), and 1(g)).

Although the relaxation induced by angiotensin II has not been altered by apocynin, tiron, PEG-catalase, and DCPIB or by the chronic treatment with angiotensin-(1–7) in endothelium-intact carotid rings from nontreated control rats, this response was completely restored by apocynin or tiron and partially restored by PEG-catalase or DCPIB in endothelium-intact carotid rings from nontreated diabetic rats. Similar to the effects of apocynin or tiron, the chronic treatment with angiotensin-(1–7) completely restored the relaxation induced by angiotensin II in endothelium-intact carotid rings from diabetic rats (Figures 1(e), 1(f), 1(h), 3(a), and 3(c)). In control rat carotid, the magnitude of phenylephrine-induced precontraction was not altered by apocinin (0.28 ± 0.019 g, *n* = 9), tiron (0.27 ± 0.022 g, *n* = 9), PEG-catalase (0.30 ± 0.023 g, *n* = 9), angiotensin-(1–7) (0.32 ± 0.031 g, *n* = 9), or DCPIB (0.31 ± 0.024 g, *n* = 9). Also, in diabetic rat carotid, phenylephrine-induced precontraction was not altered by apocinin (0.53 ± 0.039 g, *n* = 9), tiron (0.49 ± 0.035 g, *n* = 9), PEG-catalase (0.50 ± 0.049 g, *n* = 9), angiotensin-(1–7) (0.51 ± 0.042 g, *n* = 9), or DCPIB (0.50 ± 0.033 g, *n* = 9), when compared to nonpretreated diabetic rat carotid (*P* > 0.05*, one-way ANOVA, Bonferroni post-hoc*).

### 3.2. Angiotensin-(1–7)-Induced Relaxation

**Figure 2:**
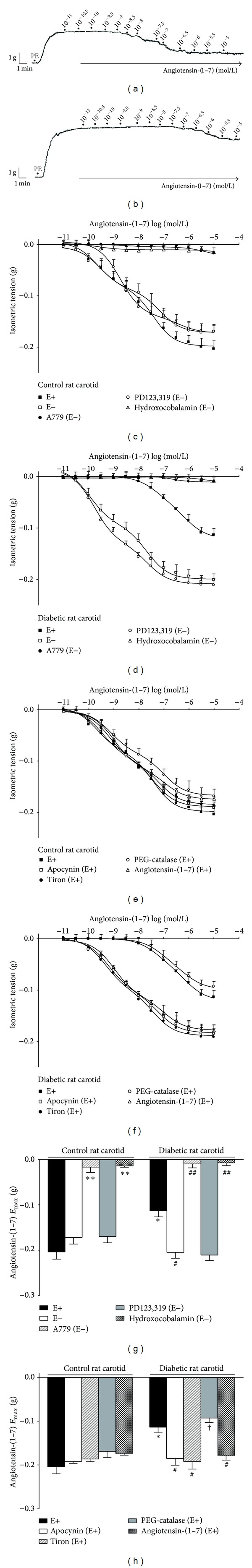
Concentration-response curves for angiotensin-(1–7) in endothelium-intact (E+) or -denuded (E−) carotid rings from control or diabetic rats, over the precontraction induced by phenylephrine (PE). Representative traces from angiotensin-(1–7)-evoked relaxation in E+ control (a) or diabetic (b) rat carotid. Effect of the* in vitro *pretreatment with A779, PD123,319 or hydroxocobalamin in carotid rings from control (c) or diabetic (d) rats. Effect of the* in vitro *pretreatment with apocynin, tiron, PEG-catalase, or the chronic (*in vivo*) treatment with angiotensin-(1–7) in carotid rings from control (e) or diabetic (f) rats. Angiotensin-(1–7) Emax in carotid from control or diabetic rats before or after the* in vitro *pretreatment with A779, PD123,319, hydroxocobalamin (g), apocynin, tiron, or PEG-catalase or the chronic (*in vivo*) treatment with angiotensin-(1–7) (h). The values are significantly different (*P* < 0.01; *n* = 9) from nonpretreated E+ (∗) or E− (∗∗) carotid rings from nontreated control rats, from nonpretreated E+ (#) or E− (##) carotid rings from nontreated diabetic rats, or from PEG-catalase pretreated E+ (†) carotid rings from nontreated control rats.

In carotid artery from nontreated control rat, angiotensin-(1–7) evoked a relaxant response (*E*_max⁡_ = −0.20 ± 0.016 g, *n* = 9) over the precontraction evoked by phenylephrine (0.30 ± 0.025 g, *n* = 9). This relaxant response was not altered by endothelium removal. In endothelium-denuded carotid rings from nontreated control rats, this relaxant response was completely inhibited by A779, hydroxocobalamin (Figures 2(a), 2(c), and 2(g)) or L-NNA (*E*_max⁡_ = −0.03 ± 0.005 g, *n* = 9) (*P* < 0.001*, one-way ANOVA, Bonferroni post-hoc*), but not altered by L-NPA (*E*_max⁡_ = −0.18 ± 0.011 g, *n* = 9) or 1400 W (*E*_max⁡_ = −0.21 ± 0.019 g, *n* = 9) (*P* > 0.05*, one-way ANOVA, Bonferroni post-hoc*). The magnitude of phenylephrine-induced precontraction in nontreated control rat carotid was not altered by endothelium removal (0.33 ± 0.031 g, *n* = 9) or the pretreatment with A779 (0.34 ± 0.028 g, *n* = 9), hydroxocobalamin (0.36 ± 0.034 g, *n* = 9), L-NNA (0.35 ± 0.031 g, *n* = 9), L-NPA (0.29 ± 0.027 g, *n* = 9), or 1400 W (0.27 ± 0.026 g, *n* = 9) (*P* > 0.05*, one-way ANOVA, Bonferroni post-hoc*).

Diabetes impaired, but not abolished, the relaxant response induced by angiotensin-(1–7) in rat carotid (*E*_max⁡_ = −0.11 ± 0.013 g) over the precontraction induced by phenylephrine (0.51 ± 0.029 g, *n* = 9). This relaxant response was completely restored by endothelium removal (over again, this result required that the characterization of the mediators of angiotensin-(1–7)-induced relaxation was performed in endothelium-denuded carotid rings from diabetic rats, as well as from control rats, to compare the responses). In endothelium-denuded carotid rings from nontreated diabetic rats, the relaxant response evoked by angiotensin-(1–7) was completely inhibited by A779, hydroxocobalamin (Figures 2(b), 2(d), and 2(g)), or L-NNA (*E*_max⁡_ = −0.01 ± 0.002 g, *n* = 9) (*P* < 0.001*, one-way ANOVA, Bonferroni post-hoc*) but not altered by L-NPA (*E*_max⁡_ = −0.12 ± 0.009 g, *n* = 9) or 1400 W (*E*_max⁡_ = −0.10 ± 0.007 g, *n* = 9) (*P* > 0.05*, one-way ANOVA, Bonferroni post-hoc*). The magnitude of phenylephrine-induced precontraction in nontreated diabetic rat carotid was not altered by endothelium removal (0.48 ± 0.035 g, *n* = 9) or the pretreatment with A779 (0.49 ± 0.025 g, *n* = 9), hydroxocobalamin (0.50 ± 0.041 g, *n* = 9), L-NNA (0.47 ± 0.023 g, *n* = 9), L-NPA (0.49 ± 0.032 g, *n* = 9), or 1400 W (0.51 ± 0.039 g, *n* = 9) (*P* > 0.05*, one-way ANOVA, Bonferroni post-hoc*).

**Figure 3:**
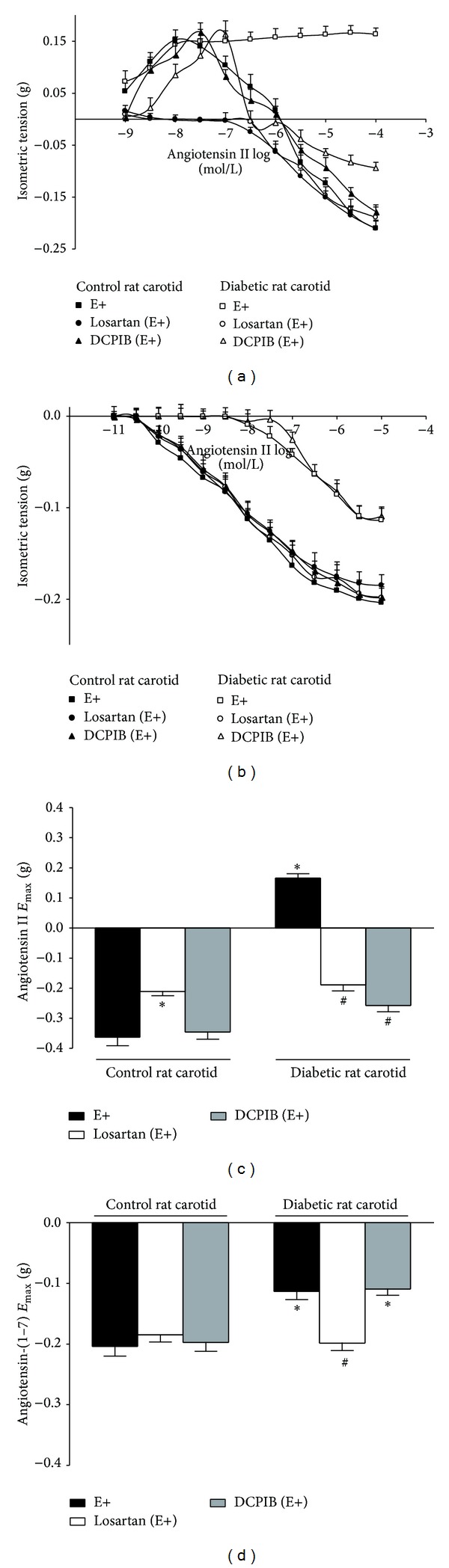
Concentration-response curves for angiotensin II or angiotensin-(1–7) in endothelium-intact (E+) carotid from control or diabetic rats after the* in vitro *pretreatment with losartan or DCPIB. Effect of the* in vitro *pretreatment with losartan or DCPIB on the relaxation induced by angiotensin II (a) or angiotensin-(1–7) (b). Angiotensin II *E*_max⁡_ (c) or angiotensin-(1–7) *E*_max⁡_ (d) before or after the* in vitro *pretreatment with losartan or DCPIB. The values are significantly different (*P* < 0.01; *n* = 9) from nonpretreated carotid rings (E+) from nontreated control (∗) or diabetic (#) rats.

In endothelium-denuded carotid rings from nontreated control or diabetic rats, the maximum relaxation induced by angiotensin-(1–7) was lesser than that one induced by angiotensin II (Figures 2(a), 2(b), and 2(e)). However, the maximum relaxation induced by angiotensin II in losartan pretreated endothelium-intact carotid rings from nontreated control or diabetic rats had the same extent as the relaxation evoked by angiotensin-(1–7) in nonpretreated endothelium-intact carotid rings from nontreated control rat (Figure 3).

Although the relaxation induced by angiotensin-(1–7) has not been altered by losartan, apocynin, or tiron in endothelium-intact carotid rings from nontreated control rats, this response was completely restored by losartan, apocynin, or tiron in endothelium-intact carotid rings from nontreated diabetic rat. However, PEG-catalase, DCPIB, or the chronic treatment with angiotensin-(1–7) did not alter the relaxation induced by angiotensin-(1–7) in endothelium-intact carotid rings from nontreated control or diabetic rats. Similarly to the effects of losartan, apocynin, or tiron, the chronic treatment with angiotensin-(1–7) completely restored the relaxation induced by angiotensin-(1–7) in endothelium-intact carotid rings from diabetic rats (Figures 2(e), 2(f), 2(h), 3(b), and 3(d)). In control rat carotid, the magnitude of phenylephrine-induced precontraction was not altered by losartan (0.27 ± 0.022 g, *n* = 9), apocinin (0.26 ± 0.023 g, *n* = 9), tiron (0.29 ± 0.027 g, *n* = 9), PEG-catalase (0.31 ± 0.028 g, *n* = 9), angiotensin-(1–7) (0.30 ± 0.027 g, *n* = 9), or DCPIB (0.29 ± 0.021 g, *n* = 9). Also, in diabetic rat carotid, phenylephrine-induced precontraction was not altered by losartan (0.48 ± 0.033 g, *n* = 9), apocinin (0.51 ± 0.042 g, *n* = 9), tiron (0.50 ± 0.044 g, *n* = 9), PEG-catalase (0.49 ± 0.036 g, *n* = 9), angiotensin-(1–7) (0.47 ± 0.035 g, *n* = 9), or DCPIB (0.52 ± 0.039 g, *n* = 9), when compared to nonpretreated diabetic rat carotid (*P* > 0.05*, one-way ANOVA, Bonferroni post-hoc*).

### 3.3. Levels of O_2_^−^

**Figure 4:**
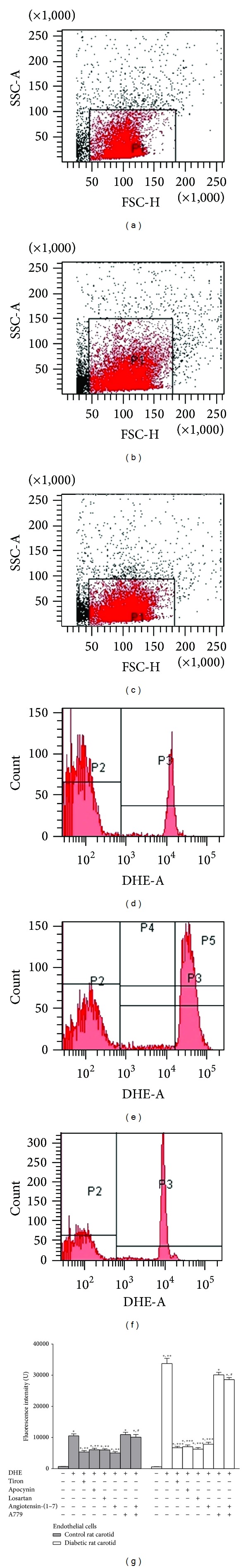
O_2_^−^ levels in carotid endothelial cells from control or diabetic rats. Dot plots show the gates of carotid endothelial cells from nontreated control rats (a) or from nontreated (b) or angiotensin-(1–7)-treated (c) diabetic rats. Histograms show the fluorescence emitted by DHE-loaded carotid endothelial cells from nontreated control rats (d) or from nontreated (e) or angiotensin-(1–7)-treated diabetic rats (f). Effects of the* in vitro *pretreatment with tiron, apocynin, or losartan or the chronic (*in vivo*) treatment with angiotensin-(1–7) combined or not with A779 on the fluorescence emitted by DHE-loaded endothelial cells samples (g). The values are significantly different (*P* < 0.01; *n* = 5) from the respective blank samples from nontreated rats (∗), from nonpretreated DHE-loaded endothelial cells samples from nontreated control (∗∗) or diabetic (∗∗∗) rats, or from the respective DHE-loaded endothelial cells samples from angiotensin-(1–7)-treated rats (#).

The FI emitted by DHE-loaded endothelial cells from carotid arteries of nontreated diabetic rats was higher than the FI emitted by control samples. Tiron, apocynin, losartan, or the chronic treatment with angiotensin-(1–7) reduced the FI emitted by DHE-loaded samples from diabetic rats to the control levels obtained in the presence of these chemicals. DCPIB did not alter the FI emitted by DHE-loaded endothelial cells from carotid arteries of nontreated control (FI = 9,758.32 ± 561.29 U, *n* = 5) or diabetic (FI = 35,094.07 ± 915.43 U, *n* = 5) rats. The antioxidant effect produced by the chronic treatment with angiotensin-(1–7) in DHE-loaded samples from both control and diabetic rats was completely inhibited by the chronic treatment with A779 (Figure 4).

### 3.4. Levels of H_2_O_2_

**Figure 5:**
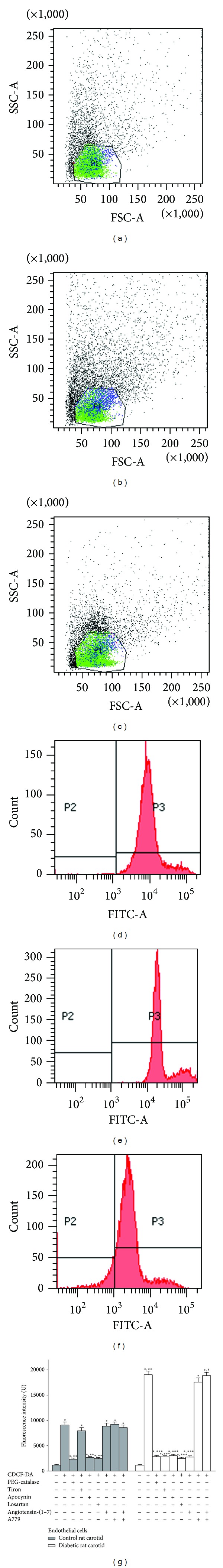
H_2_O_2_ levels in carotid endothelial cells from control or diabetic rats. Dot plots show the gates of carotid endothelial cells from nontreated control rats (a) or from nontreated (b) or angiotensin-(1–7)-treated diabetic rats (c). Histograms show the fluorescence emitted by CDCF-DA-loaded carotid endothelial cells from nontreated control rats (d) or from nontreated (e) or angiotensin-(1–7)-treated diabetic rats (f). Effects of the* in vitro *pretreatment with PEG-catalase, tiron, apocynin, or losartan or the chronic (*in vivo*) treatment with angiotensin-(1–7) combined or not with A779 on the fluorescence emitted by CDCF-DA-loaded endothelial cells samples (g). The values are significantly different (*P* < 0.01; *n* = 5) from the respective blank samples from nontreated rats (∗), from nonpretreated CDCF-DA-loaded endothelial cells samples from nontreated control (∗∗) or diabetic (∗∗∗) rats, or from CDCF-DA-loaded endothelial cells samples from angiotensin-(1–7)-treated diabetic rats (#).

The FI emitted by CDCF-DA-loaded endothelial cells from carotid arteries of nontreated diabetic rats was higher than the FI emitted by control samples. In CDCF-DA-loaded endothelial cells from carotid arteries of nontreated diabetic rats, PEG-catalase, tiron, apocynin, losartan, or the chronic treatment with angiotensin-(1–7) reduced the FI to the control levels obtained in the presence of PEG-catalase, apocynin, or losartan. DCPIB did not alter the FI emitted by CDCF-DA-loaded endothelial cells from carotid arteries of nontreated control (FI = 10,322.04 ±517.81 U, *n* = 5) or diabetic (FI = 21,085.01 ±639.43 U, *n* = 5) rats. The antioxidant effect produced by the chronic treatment with angiotensin-(1–7) in CDCF-DA-loaded samples from diabetic rats was completely inhibited by the chronic treatment with A779 (Figure 5).

### 3.5. ACE2, Angiotensin-(1–7), and* Mas*-Receptors Expression

**Figure 6:**
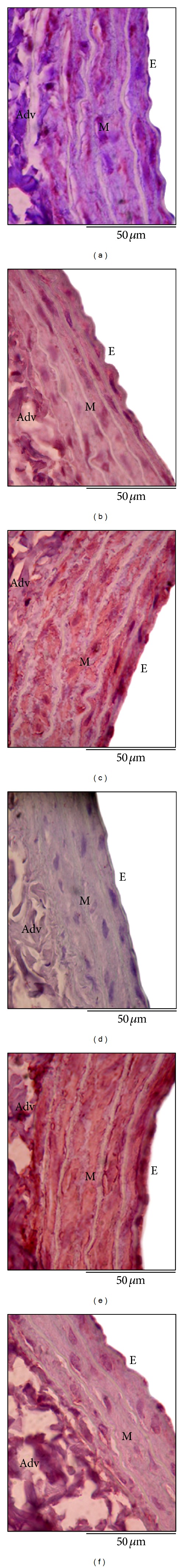
ACE2, angiotensin-(1–7) and* Mas* receptors staining in carotid arteries from nontreated control or diabetic rats. ACE2 expression in nontreated control (a) or diabetic (b) rat carotid, angiotensin-(1–7) levels in nontreated control (c) or diabetic (d) rat carotid, and* Mas* receptors expression in nontreated control (e) or diabetic (f) rat carotid. E: endothelium; M: media; Adv: adventitia. The immunostaining is denoted in red (magnification: 100x).

In the muscular layer from nontreated diabetic rat carotid, ACE2 staining (39.05 ± 3.42%) was not altered, while angiotensin-(1–7) (2.09 ± 1.22%) or* Mas* receptors (17.63 ± 1.62%) staining was reduced when compared to nontreated control rat carotid (ACE2 = 47.28 ± 4.51%; angiotensin-(1–7) = 81.04 ± 6.29%; *Mas* = 92.37 ± 7.24%).* Mas* receptors staining was also detected in the endothelium from nontreated control rat carotid (64.57 ± 6.92%), but it was reduced in the endothelium from nontreated diabetic rat carotid (38.01 ± 2.79%) (*P* < 0.001, *n* = 5*, one-way ANOVA, Bonferroni post-hoc*) (Figure 6).

The chronic treatment with angiotensin-(1–7) did not alter ACE2 expression in the muscular layer from control (42.37 ± 3.91%) or diabetic rat carotid (41.25 ± 4.14%). The chronic treatment with DX600 reduced angiotensin-(1–7) staining in the muscular layer from carotid arteries of control rats (4.01 ± 3.17%) to the levels obtained in carotid arteries from nontreated diabetic rats. On the other hand, the chronic treatment with DX600 did not alter angiotensin-(1–7) staining in the muscular layer from carotid arteries of diabetic rats (2.85 ± 1.36%) when compared to nontreated diabetic rats (*P* < 0.001, *n* = 5*, one-way ANOVA, Bonferroni post-hoc*).

The chronic treatment with angiotensin-(1–7) did not alter the expression of* Mas* receptors neither in the endothelium from control (59.87 ± 5.76%) or diabetic (34.21 ± 3.07%) rat carotid nor in the muscular layer from control (87.23 ± 8.19%) or diabetic (14.95 ± 1.24%) rat carotid when compared to the carotid arteries from the respective nontreated groups (*P* < 0.001, *n* = 5*, one-way ANOVA, Bonferroni post-hoc*).

### 3.6. Blood Pressure, Carotid Blood Flow, and Carotid Resistance

Blood pressure was not different between nontreated or treated control or diabetic rats. In nontreated diabetic rats, carotid blood flow was reduced and carotid resistance was increased when compared to nontreated control rats. The chronic treatment of control rats with DX600 significantly reduced carotid blood flow and increased carotid resistance to the levels obtained in nontreated diabetic rats. The same effect was observed in carotid blood flow and carotid resistance from control rats that were treated with A779. In diabetic rats, carotid blood flow or carotid resistance was not altered by the chronic treatment with DX600 or A779. The chronic treatment with angiotensin-(1–7) did not alter carotid blood flow or carotid resistance from control rats, but it restored carotid blood flow and carotid resistance from diabetic rats. A779 inhibited the effect of the chronic treatment with angiotensin-(1–7) on carotid blood flow and carotid resistance from diabetic rats (Table 3).

## 4. Discussion

The present study has three major new findings. The first one shows that type I-diabetes impairs the functionality of ACE2-angiotensin-(1–7)-*Mas* axis in rat carotid by a mechanism that involves the endothelial AT_1_-activated NAD(P)H oxidase-driven generation of O_2_^−^ and H_2_O_2_: while H_2_O_2_ derived from O_2_^−^ dismutation inhibits ACE2 activity in generating angiotensin-(1–7) seemingly by activating *I*_Cl,SWELL_, O_2_^−^ inhibits the nitrergic vasorelaxant effect evoked by angiotensin-(1–7) upon* Mas* receptors activation. The second new finding shows that the impaired functionality of ACE2-angiotensin-(1–7)-*Mas* axis increases carotid resistance, which reduces carotid blood flow during type I-diabetes, highlighting the importance of the vasoprotective role assigned to the activation of this axis in both healthy and diseased conditions. Finally, the third new finding shows that the chronic treatment with angiotensin-(1–7) restores the functionality of carotid ACE2-angiotensin-(1–7)-*Mas* axis, what contributes to restore the carotid resistance and blood flow in diabetic conditions, by a mechanism that involves* Mas*-mediated antioxidant effects against the endothelial generation of O_2_^−^ and H_2_O_2_ catalyzed by AT_1_-activated NAD(P)H oxidase.

The functionality of vascular ACE2 (i.e., the activity of vascular ACE2 in generating angiotensin-(1–7)) was studied by relaxant curves induced by angiotensin II in rat carotid. This approach is supported by the evidences that angiotensin II-evoked relaxation in rat carotid is partially mediated by* Mas* receptors [19], seemingly upon angiotensin II hydrolysis into angiotensin-(1–7) and that the only enzyme able to convert angiotensin II into angiotensin-(1–7) in vascular tissues is ACE2 [17, 25]. Our findings show that angiotensin II-evoked relaxation curve in rat carotid is characterized by a biphasic response consisting in a residual AT_1_-mediated contraction induced by nanomolar concentrations of angiotensin II (about 0.15 g), followed by an endothelium-independent relaxation induced by micromolar concentrations of this peptide (about −0.35 g). Our findings also show that DX600 completely abolishes the relaxation induced by angiotensin II without changing the residual contraction mediated by AT_1_-receptors, which allows us to conclude that the relaxant response induced by angiotensin II in rat carotid occurs upon the ACE2-catalyzed hydrolysis of angiotensin II into angiotensin-(1–7). In turn, we found that losartan or A779 partially inhibits the relaxation triggered by angiotensin II: while losartan abrogates the complete relaxation of the residual contraction evoked by angiotensin II by inhibiting this contractile response, A779 does not alter neither the residual contraction evoked by angiotensin II nor the relaxation of this contractile response but abrogates the relaxation beyond the relaxant response that counteracts the residual contraction induced by angiotensin II. Thus, these findings allow us to divide the ACE2-dependent relaxation induced by angiotensin II in rat carotid into two components: (1) the losartan-sensitive relaxant response (about −0.15 g), which occurs upon the dissociation of angiotensin II from AT_1_-receptors and the subsequent ACE2-catalyzed hydrolysis of angiotensin II into angiotensin-(1–7) and (2) the A779-sensitive relaxant response (about −0.2 g), which depends on the activation of* Mas *receptors by angiotensin-(1–7) generated from angiotensin II. Indeed, the vascular generation of angiotensin-(1–7) from angiotensin II is catalyzed by ACE2, which hydrolyzes only angiotensin II with high catalytic efficiency among the angiotensin peptides [17, 25]. Moreover, Tirapelli et al. [19] had already shown that* Mas* receptors partially mediate angiotensin II-induced relaxation in rat carotid.

Our results show that, similar to angiotensin II, angiotensin-(1–7) also evokes an endothelium-independent relaxation (about −0.2 g), which is completely mediated by* Mas*-receptors, NO, and endothelial NOS (*e*NOS) in rat carotid. In agreement with these findings, we also show that* Mas *receptors are mostly expressed in the muscular layer from rat carotid, supporting our functional evidence concerning the* Mas*-mediated endothelium-independent relaxation evoked by angiotensin-(1–7) in this vessel. Indeed, the vasorelaxation mediated by* Mas *receptors has mostly been described as a NOS-dependent nitrergic mechanism [19, 41–44], since the activation of these receptors by angiotensin-(1–7) leads to the phosphorylation of* e*NOS [45], which is also expressed in smooth muscle layer from rat carotid [9]. Another important aspect from our results is that the relaxant response induced by angiotensin-(1–7) had the same extent of the* Mas*-mediated (i.e., the A779-sensitive) relaxation evoked by angiotensin II in rat carotid, confirming that part of the relaxation induced by angiotensin II in this vessel is mediated by angiotensin-(1–7) upon the ACE2-catalyzed hydrolysis of the octapeptide.

Interestingly, our findings show that type I-diabetes inhibits the relaxant response induced by angiotensin II but only attenuates the* Mas*-mediated endothelium-independent relaxation evoked by angiotensin-(1–7) in rat carotid. Moreover, we observed that the residual relaxation evoked by angiotensin-(1–7) in diabetic rat carotid is mediated by NO and* e*NOS. Although the muscular expression of* e*NOS in this vessel is significantly reduced [9], recent evidences have shown that the acute stimulation with nano- to micromolar concentrations of angiotensin-(1–7) increases* e*NOS expression in rat cerebral ischemic tissues [46], spontaneously hypertensive rat ventricles [47], and human circulating fibrocytes [48]. These findings allow us to suggest that the generation of NO from muscular* e*NOS in diabetic rat carotid during angiotensin-(1–7)-evoked relaxation may be related to one of the following reasons: (1) the residual expression of* e*NOS at the muscular layer form diabetic rat carotid is enough to generate the NO levels that mediate this relaxant response or (2) the acute stimulation with nano- to micromolar concentrations of angiotensin-(1–7) may increase the expression of* e*NOS in this vessel, what would support the NO generation during the relaxation.

According to the present results, the inhibition of angiotensin II-induced relaxation in diabetic rat carotid, taken together with the findings concerning the unchanged muscular expression of ACE2 and the reduced muscular levels of angiotensin-(1–7) in this vessel, allows us to strongly suggest that diabetes impairs the ACE2-catalyzed hydrolysis of angiotensin II into angiotensin-(1–7), but not the expression of the carboxypeptidase. This finding is confirmed when we show that the endogenous generation of angiotensin-(1–7) in rat carotid, which is catalyzed by ACE2, is lost in diabetic rats, since the chronic treatment with DX600 reduced angiotensin-(1–7) levels only in carotid arteries from normoglycaemic animals. Moreover, our functional results show that the diabetes-elicited inhibition of ACE2-catalyzed hydrolysis of angiotensin II in rat carotid (and thus, the inhibition of angiotensin II-induced relaxation) involves endothelial pathways completely mediated by AT_1_-receptors, NAD(P)H oxidase, and O_2_^−^ but partially mediated by H_2_O_2_. In turn, diabetes-elicited impairment of angiotensin-(1–7)-evoked relaxation involves endothelial mechanisms that are only mediated by AT_1_-receptors, NAD(P)H oxidase, and O_2_^−^, but not by H_2_O_2_. In addition to these findings, we also observed that the* Mas*-mediated (A779-sensitive) relaxation induced by angiotensin II in PEG-catalase pretreated carotid rings from diabetic rat had the same extent as the relaxation induced by angiotensin-(1–7) in diabetic rat carotid (i.e., about −0.1 g). Taken together, these findings allow us to conclude that H_2_O_2_ generated from endothelial AT_1_-activated NAD(P)H oxidase-derived O_2_^−^ inhibits the ACE2-catalyzed hydrolysis of angiotensin II into angiotensin-(1–7), while AT_1_-activated NAD(P)H oxidase-derived O_2_^−^ impairs the nitrergic transduction pathways underlying* Mas* receptors activation in diabetic rat carotid. Confirming these data, we found an increase in the levels of H_2_O_2_ produced from AT_1_-activated NAD(P)H oxidase-derived O_2_^−^, whose generation was also increased in endothelial cells from diabetic rat carotid. Indeed, type I-diabetes increases the endothelial generation of O_2_^−^ derived from NAD(P)H oxidase in rat carotid [8, 9]. Moreover, the generation of H_2_O_2_ from O_2_^−^ can occur by the dismutation of O_2_^−^ [49]. In turn, upon AT_1_-receptors activation by angiotensin II in endothelial cells, Nox1 subunit from vascular NAD(P)H oxidase can generate O_2_^−^, which is latter converted to H_2_O_2_ [50, 51]. Finally, type I-diabetes enhances the endothelial expression of AT_1_-receptors and the reactive oxygen species generation mediated by these receptors in rat carotid, what explains the selective activation of endothelial NAD(P)H oxidase by AT_1_-receptors in this vessel [8].

The cleavage of angiotensin II by somatic ACE2 can be reduced by increasing the physiological extracellular levels of Cl^−^ [30], due to the presence of a regulatory Cl^−^ binding site at the extracellular region of the carboxypeptidase [52]. Added to these findings, H_2_O_2_ generated from NAD(P)H oxidase-derived O_2_^−^ can activate *I*_Cl,SWELL_ in vascular smooth muscle cells, leading to the efflux of Cl^−^ and thus increasing Cl^−^ extracellular levels [31, 32]. Taken together, these findings prompted us to investigate the role of *I*_Cl,SWELL_ in the modulation of ACE2-catalyzed hydrolysis of angiotensin II into angiotensin-(1–7) during the relaxation evoked by angiotensin II in diabetic rat carotid. Interestingly, we observed that the inhibition of *I*_Cl,SWELL_ partially restored the relaxation evoked by angiotensin II, but not by angiotensin-(1–7), in diabetic rat carotid, similarly to the effect produced by the removal of H_2_O_2_. In addition, by showing that DCPIB did not alter O_2_^−^ or H_2_O_2_ levels in endothelial cells from diabetic rat carotid, we excluded the hypothesis that the inhibition of *I*_Cl,SWELL_ could lead to the scavenging of reactive oxygen species that modulate angiotensin II-evoked relaxation in this vessel. Thus, our data, supported by the findings from Rushworth et al. [30], Ren et al. [31], Matsuda et al. [32], and Towler et al. [52], strongly suggest that H_2_O_2_ generated from endothelial AT_1_-activated NAD(P)H oxidase-derived O_2_^−^ inhibits the ACE2-catalyzed hydrolysis of angiotensin II into angiotensin-(1–7) seemingly by activating *I*_Cl,SWELL_ in smooth muscle cells from diabetic rat carotid. In turn, O_2_^−^ derived from endothelial AT_1_-activated NAD(P)H oxidase can impair nitrergic signaling pathways during type I-diabetic conditions, like the* Mas*-mediated relaxation evoked by angiotensin-(1–7) in rat carotid, by converting NO into peroxynitrite (ONOO^−^) or by inducing to NOS uncoupling [53].

One of the most important aspects from our results is that the chronic treatment with angiotensin-(1–7) restores the functionality of ACE2-Angiotensin-(1–7)-*Mas* axis (i.e., both angiotensin II- and angiotensin-(1–7)-evoked relaxation) in diabetic rat carotid. According to our findings, this protective effect results from a* Mas*-mediated mechanism that inhibits the generation of O_2_^−^ and H_2_O_2_ catalyzed by AT_1_-activated NAD(P)H oxidase in endothelial cells from diabetic rat carotid. Moreover, we excluded the hypothesis that the restoration of ACE2-Angiotensin-(1–7)-*Mas* axis functionality in carotid arteries from angiotensin-(1–7)-treated diabetic rats could be resultant from a translational effect of enhancing ACE2 or* Mas* receptors expression, since they remained unaltered after the treatment. Thus, our results can be explained by the findings from Sampaio et al. [21], who described that the activation of* Mas *receptors by angiotensin-(1–7) inhibits the AT_1_-mediated NAD(P)H oxidase assembly and the subsequent reactive oxygen species generation upon angiotensin II stimulation in endothelial cells.

Interestingly, even if type I-diabetes subregulates the endothelial expression of* Mas *receptors in rat carotid, the activation of this residual population of receptors during the chronic treatment with* Mas* agonists can trigger antioxidant effects that are enough to overcome the impairment of the local ACE2-angiotensin-(1–7)-*Mas* axis functionality. These findings have important implications to the therapeutic efficacy of* Mas* agonists on attenuating vascular dysfunctions and complications, since we showed that the activation of* Mas *receptors triggers a positive feedback over the ACE2-angiotensin-(1–7)-*Mas* axis when the functionality of this vasoprotective axis is impaired, even during diseases that subregulate the vascular expression of these receptors, such as type I-diabetes. Indeed, the chronic treatment of type I-diabetic rats with angiotensin-(1–7) or the nonpeptide* Mas* agonist AVE-0991 was efficacious in restoring the contractile and relaxation functions of mesenteric bed, carotid, and renal arteries [20].

Finally, we found that the chronic treatment with DX600 or A779 reduced carotid blood flow by increasing carotid resistance in normoglycaemic rats to the levels obtained with nontreated diabetic rats. On the other hand, carotid resistance and blood flow in diabetic rats were not altered after the chronic treatment with DX600 or A779. Moreover, while the chronic treatment with angiotensin-(1–7) did not alter carotid resistance and blood flow in normoglycaemic rats, it restored these parameters in diabetic rats, and this protective effect of angiotensin-(1–7) was inhibited when angiotensin-(1–7) was coadministrated with A779. Taken together, these findings point the vasoprotective role assigned to the ACE2-angiotensin-(1–7)-*Mas* axis functionality in maintaining carotid resistance and blood flow and confirm that the impairment of the functionality of this axis damages carotid function in diabetic conditions. Considering that carotid blood flow rate is determined, in a higher extent, by the resistance of smaller proximal arteries and arterioles (such as internal and external carotid arteries) than the carotid resistance, our findings allow us to suggest that the impairment of ACE2-angiotensin-(1–7)-*Mas* axis functionality reaches not only carotid artery but also the proximal resistance vessels.

## 5. Conclusions

**Figure 7:**
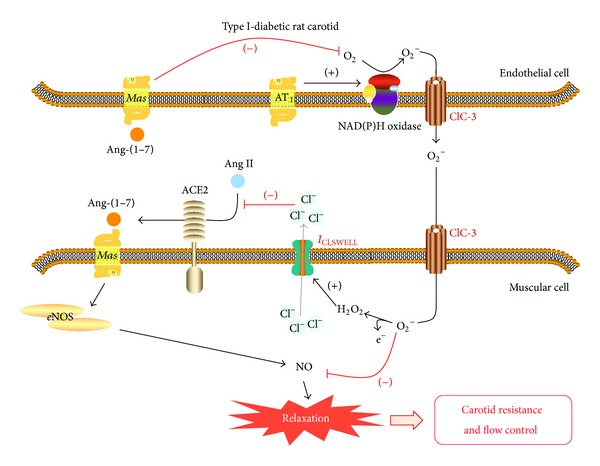
Conclusive graphical abstract. Endothelial AT_1_-activated NAD(P)H oxidase-driven generation of O_2_^−^ and H_2_O_2 _in carotid arteries from type I-diabetic rats impairs the functionality of the local vasoprotective ACE2-angiotensin-(1–7)-*Mas* axis, which in turn impairs carotid blood flow. In this mechanism, H_2_O_2_ derived from O_2_^−^ dismutation inhibits ACE2 activity in generating angiotensin-(1–7) by activating *I*_Cl,SWELL_, while O_2_^−^ inhibits the nitrergic vasorelaxant effect evoked by angiotensin-(1–7) upon* Mas* receptors activation. The chronic treatment of diabetic rats with angiotensin-(1–7) restores the functionality of carotid ACE2-angiotensin-(1–7)-*Mas* axis by triggering a positive feedback on this axis, played by a residual population of endothelial* Mas*-receptors that blunts the endothelial AT_1_-activated NAD(P)H oxidase-driven generation of reactive oxygen species in rat carotid.* Mas*-mediated antioxidant effects evoked by the chronic treatment with angiotensin-(1–7) also restores carotid resistance and blood flow in diabetic rats, pointing the important contribution of the ACE2-angiotensin-(1–7)-*Mas* axis in maintaining carotid function.

This is the first study that shows that the endothelial AT_1_-activated NAD(P)H oxidase-driven generation of O_2_^−^ and H_2_O_2_ in carotid arteries from type I-diabetic rats impairs the functionality of the local vasoprotective ACE2-angiotensin-(1–7)-*Mas* axis, which in turn impairs carotid blood flow. In this mechanism, H_2_O_2_ derived from O_2_^−^ dismutation inhibits ACE2 activity in generating angiotensin-(1–7) seemingly by activating *I*_Cl,SWELL_, while O_2_^−^ inhibits the nitrergic vasorelaxant effect evoked by angiotensin-(1–7) upon* Mas* receptors activation. Furthermore, we originally showed that the chronic treatment of diabetic rats with angiotensin-(1–7) restores the functionality of carotid ACE2-angiotensin-(1–7)-*Mas* axis by triggering a positive feedback on this axis, played by a residual population of endothelial* Mas*-receptors that blunts the endothelial AT_1_-activated NAD(P)H oxidase-driven generation of reactive oxygen species in rat carotid. Finally, we found that the* Mas*-mediated antioxidant effects evoked by the chronic treatment with angiotensin-(1–7) also restores carotid resistance and blood flow in diabetic rats, pointing the important contribution of the ACE2-angiotensin-(1–7)-*Mas* axis in maintaining carotid function (Figure 7). These findings have relevant implications to support the therapeutic efficacy of* Mas* agonists on preventing or attenuating diabetic endothelial dysfunction in carotid arteries and its underlying cerebrovascular complications by enhancing the vasoprotective role of the local ACE2-angiotensin-(1–7)-*Mas* axis.

## Figures and Tables

**Table 1:** Fasting blood glucose levels from control or diabetic rats.

Rats	Glycaemia (mg·dL^−1^)	
	Day 0	Day 2
Nontreated Control	68.7 ± 3.11	73.0 ± 6.35
Nontreated Diabetic	75.4 ± 5.17	392.6 ± 11.47^∗,#^
DX600-treated control	70.5 ± 4.20	71.8 ± 5.23
DX600-treated diabetic	69.9 ± 6.31	402.7 ± 15.25^∗,#^
Angiotensin-(1–7)-treated control	73.1 ± 5.19	67.9 ± 5.24
Angiotensin-(1–7)-treated diabetic	71.2 ± 4.98	385.9 ± 13.26^∗,#^
A779-treated control	72.1 ± 4.57	76.3 ± 7.93
A779-treated diabetic	74.2 ± 7.21	406.0 ± 14.69^∗,#^
Angiotensin-(1–7) + A779-treated control	67.5 ± 6.52	69.3 ± 6.45
Angiotensin-(1–7) + A779-treated diabetic	71.2 ± 7.09	409.1 ± 17.33^∗,#^

The values are significantly different (*P* < 0.01; *n* = 11) from the respective control rats at the same day (∗) or the same rats at day 0 (#).

**Table 2:** Body weight from control or diabetic rats.

Rats	Body weight (g)	
	Day 0	Day 42
Nontreated control	374.9 ± 7.5	714.1 ± 10.2^#^
Nontreated diabetic	365.2 ± 6.9	286.3 ± 9.1^∗,#^
DX600-treated control	361.5 ± 5.11	721.2 ± 11.4^#^
DX600-treated diabetic	378.0 ± 4.9	267.5 ± 10.3^∗,#^
Angiotensin-(1–7)-treated control	373.8 ± 6.2	733.9 ± 8.7^#^
Angiotensin-(1–7)-treated diabetic	354.2 ± 8.2	275.3 ± 11.6^∗,#^
A779-treated control	369.1 ± 5.7	729.4 ± 7.5^#^
A779-treated diabetic	372.4 ± 9.1	281.6 ± 13.2^∗,#^
Angiotensin-(1–7) + A779-treated control	368.4 ± 7.2	719.1 ± 9.5^#^
Angiotensin-(1–7) + A779-treated diabetic	355.7 ± 6.9	272.3 ± 11.4^∗,#^

The values are significantly different (*P* < 0.01; *n* = 11) from the respective control rats at the same day (∗) or the same rats at day 0 (#).

**Table 3:** Blood pressure (BP), carotid blood flow (*F*), and carotid resistance (*R*) from control or diabetic rats.

Rats	Cardiovascular parameters		
	BP (mmHg)	*F* (mL/min)	*R* (U)
Nontreated control	81.7 ± 5.4	6.11 ± 0.48	14.1 ± 2.3
Nontreated diabetic	99.2 ± 8.1	2.37 ± 0.21*	44.6 ± 3.2*
DX600-treated control	76.4 ± 6.2	2.09 ± 0.17*	41.9 ± 3.5*
DX600-treated diabetic	89.2 ± 7.5	2.13 ± 0.19	45.2 ± 4.2
Angiotensin-(1–7)-treated control	79.5 ± 6.1	5.76 ± 0.32	13.2 ± 2.9
Angiotensin-(1–7)-treated diabetic	97.0 ± 9.1	6.29 ± 0.51^#^	15.0 ± 2.1^#^
A779-treated control	81.1 ± 7.4	1.99 ± 0.14*	40.2 ± 4.3*
A779-treated diabetic	98.7 ± 8.3	2.44 ± 0.23	42.4 ± 4.1
Angiotensin-(1–7) + A779-treated control	75.6 ± 7.0	2.05 ± 0.19^∗,†^	38.2 ± 3.8^∗,†^
Angiotensin-(1–7) + A779-treated diabetic	99.5 ± 9.2	1.97 ± 0.11^†^	46.3 ± 4.7^†^

The values are significantly different (*P* < 0.01; *n* = 5) from nontreated control rats (∗), nontreated diabetic rats (#), or the respective rats treated with angiotensin-(1–7) (^†^).
